# Mitochondrial ClpP serine protease-biological function and emerging target for cancer therapy

**DOI:** 10.1038/s41419-020-03062-z

**Published:** 2020-10-09

**Authors:** Kazem Nouri, Yue Feng, Aaron D. Schimmer

**Affiliations:** 1 Princess Margaret Cancer Centre, University Health Network, Toronto, ON Canada; 2 Department of Medical Biophysics, University of Toronto, Toronto, ON Canada

**Keywords:** Drug development, Translational research

## Abstract

Mitochondrial ClpP is a serine protease located in the mitochondrial matrix. This protease participates in mitochondrial protein quality control by degrading misfolded or damaged proteins, thus maintaining normal metabolic function. Mitochondrial ClpP is a stable heptamer ring with peptidase activity that forms a multimeric complex with the ATP-dependent unfoldase ClpX (ClpXP) leading to proteolytic activity. Emerging evidence demonstrates that ClpXP is over-expressed in hematologic malignancies and solid tumors and is necessary for the viability of a subset of tumors. In addition, both inhibition and hyperactivation of ClpXP leads to impaired respiratory chain activity and causes cell death in cancer cells. Therefore, targeting mitochondrial ClpXP could be a novel therapeutic strategy for the treatment of malignancy. Here, we review the structure and function of mitochondrial ClpXP as well as strategies to target this enzyme complex as a novel therapeutic approach for malignancy.

## Facts

- ClpP forms a proteolytic complex with the AAA + chaperon ClpX termed ClpXP.
- ClpXP maintains protein quality control in the mitochondria by degrading denatured or misfolded proteins.
- A subset of primary samples from patients with hematologic malignancies and solid tumors have increased ClpXP expression compared to normal tissues.
- Mitochondrial ClpXP is essential for the viability of a subset of hematologic malignancies and solid tumors.
- Unique to this protease, both inhibition and hyperactivation of ClpP impairs oxidative phosphorylation and have anticancer effects.

## Open questions

- How does ClpXP recognize proteins for degradation?
- Why does inhibiting ClpXP kill malignant cells, but not normal cells?
- How do malignant cells become resistant to inhibition and activation of ClpXP?
- How does the expression of ClpXP change at relapse after chemotherapy?
- Can ClpP inhibitors be advanced to clinical trials?
- Will activators of ClpP demonstrate sufficient clinical efficacy?

## Introduction

Caseinolytic peptidase P (ClpP) proteolytic complex is a multimeric serine protease found in many prokaryotes and the mitochondria of eukaryotic cells and chloroplasts^1,2^. This peptidase complex has been comprehensively studied in bacteria, while its role in mammalian mitochondrial is less understood^2–4^. In bacteria, inhibition or hyperactivation of ClpP is a novel antimicrobial strategy to target drug-resistant bacteria. In addition, recent data also suggest that targeting mitochondrial ClpP could be an effective anticancer strategy for malignancies such as acute myeloid leukemia.

Mitochondria are intracellular double membrane organelles responsible for the conversion of energy-carrying molecules into ATP through the process of oxidative phosphorylation (OXPHOS)^5,6^. In addition to energy production, mitochondria regulate many other critical cellular functions such as reactive oxygen species (ROS) generation, calcium flux, macromolecule biogenesis (i.e., protein and nucleic acids), lipid synthesis, regulation of apoptosis, and antioxidant protection^7,8^.

Mitochondria contain their own genetic information, termed mitochondrial DNA (mtDNA) which is ~16.7 kb and encodes 13 mitochondrial proteins that constitute essential subunits within the respiratory chain. All respiratory chain complexes, except respiratory chain complex II, have protein subunits that are encoded by mitochondrial DNA^9–11^. While mitochondria encode for 13 proteins, the remaining 99% of mitochondrial proteins are encoded by nuclear genes, which are translated in the cytosol and imported into the mitochondria through targeting sequences^9,12^. The abundance of mitochondrial proteins depends on the transcription, RNA processing, translation efficiency, protein stability, and efficiency of mitochondrial targeting^12^.

Mitochondria have multiple mechanisms to maintain optimal protein structure and function, including the proper folding of newly imported proteins and the degradation of damaged and misfolded ones. Maintaining mitochondrial protein homeostasis is mediated by specialized molecular chaperones and proteases^9,13,14^.

Degradation of damaged proteins is an important component of mitochondrial protein quality control. Mitochondria harbor an independent proteolytic system comprising of at least 45 proteases localized throughout the different compartments of human mitochondria including the outer membrane, intermembrane space, inner membrane, and mitochondrial matrix^15^. Of these, 23 are located exclusively in the mitochondria, and others shuttle between the cytosol and mitochondria^15,16^. Five of these 23 mitochondria-localized enzymes are pseudomitoproteases with no catalytic activity but function as subunits of proteolytic complexes. The remaining 18 intrinsic mitoproteases can be classified as ATP-dependent peptidases, processing peptidases, oligo peptidases, and other mitochondrial peptidases (Table 1)^15,16^.

**Table 1:** Intrinsic mitochondrial proteases and functions.

Category	Symbol	Class	Localization	Functions	Reference(s)
	CLPP	Ser	Matrix	Protein quality control transcription/Translation ribosome assembly	^25,95,96^
ATP-dependent proteases	LONP1	Ser	Matrix	Protein quality control Mitochondrial biogenesis mtDNA maintainence mtDNA replication Adaptation to hypoxia	^25,96–98^
	AFG3L2 AFG3L2/SPG7	Metallo	Matrix/IM	Protein quality control Mitochondrial biogenesis Ribosome assembly MCU assembly	^15,25,99^
	YME1L (FTSH1)	Metallo	IM/IMS	Protein quality control Mitochondrial biogenesis Protein import Lipid trafficking Mitochondrial dynamics	^17,100,101^
	ATP23	Metallo	IMS	Protein quality control Protein maturation F1FO-ATP synthase assembly	^102,103^
	IMMP1L IMMP2L	Ser	IM/IMS	Protein maturation Apoptosis/senescence	^104,105^
	METAP1D	Metallo	Matrix	Protein import and activation	^15,25,106^
Processing peptidases	MIP	Metallo	Matrix	Coenzyme Q biosynthesis Complex III and IV activity Protein import and activation	^15,107,108^
	OMA1	Metallo	IMS/IM	Mitochondrial dynamics mitophagy and apoptosis	^17^ ^25^
	PARL	Ser	IM	Mitophagy and apoptosis Coenzyme Q biosynthesis Complex III assembly Lipid trafficking	^109^ ^110^ ^111,112^
	PMPCB	Metallo	Matrix	Protein maturation	^15,113^
	XPNPEP3	Metallo	Matrix	Protein import and activation Protein stability	^114^
Oligopeptidases	MEP	Metallo	IMS	Protein quality control	^15^ ^25^
	PITRM1	Metallo	Matrix	Protein quality control	^15^ ^25^
Other mitochondrial proteases	HTRA2 (OMI)	Ser	IMS	Protein quality control mitophagy and apoptosis Stress signaling Cristae structure maintenance	^115,116^ ^18^
	LACTB	Ser	IMS	Mitochondrial biogenesis PE metabolism	^117^ ^25^

*IM* inner membrane, *IMS* intermembrane space, *MCU* mitochondrial Ca2+ uniporter, *PE* phosphatidylethanolamine.

For example, OMA1 (Metalloendopeptidase OMA1) is a processing peptidase located in the mitochondrial inner membrane and intermembrane space. OMA1 cleaves the inner mitochondrial protein OPA1(Dynamin-like 120 kDa protein) to regulate mitochondrial dynamics. Upon loss of mitochondrial membrane potential, OMA1 cleaves OPA1, resulting in OPA1 inactivation and decreased mitochondrial fusion^17^. High temperature requirement peptidase 2 (HTRA2) (also called OMI) is another protease in the mitochondrial intermembrane space, which plays a critical role in maintaining mitochondrial cristae structure by interacting and degrading its substrate in the mitochondrial intermembrane space bridging (MIB) complex, inner membrane mitochondrial protein (IMMT)^18^. HTRA2 is also released into the cytoplasm during apoptosis where it binds and inhibits Baculoviral IAP Repeat Containing (BIRC) proteins (also called inhibitor of apoptosis proteins, IAPs), leading to an increase in caspase activity^19,20^.

**Fig. 1:**
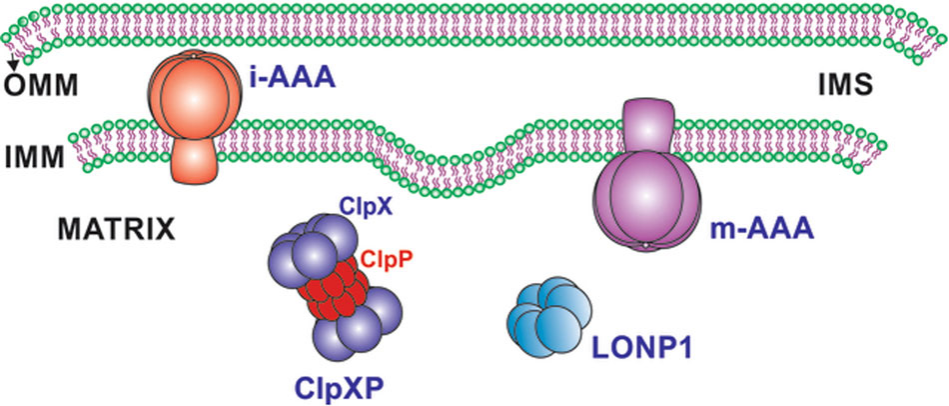
Schematic representation of ATP-dependent proteases. Mammalian mitochondria contains four proteases of the AAA+ superfamily to modulate protein quality control. The Lon protease 1, and ClpXP complex in the matrix and the i-AAA, m-AAA proteases in IM. OMM outer mitochondrial membrane, IMS intermembrane space, IMM inner mitochondrial membrane.

Among these proteases, the ATP-dependent proteases are active in all mitochondrial compartments and represent core components of the mitochondrial proteolytic system performing both quality control and regulatory functions^13,21^. The members of this family are the Lon protease localized to the mitochondrial matrix, the homologous i-AAA, and m-AAA proteases localized to the inner mitochondrial membrane, and the ClpXP complex localized to the mitochondrial matrix (the serine protease ClpP and the AAA+ATPase ClpX) (Fig. 1)^10,22–24^. These proteases degrade inner membrane proteins including subunits of respiratory complexes and translocases, as well as proteins within the matrix, intermembrane space, and outer membrane.

This review focuses on the mitochondrial ClpP protease and its regulatory subunit ClpX (referred to as the ClpXP complex) that reside in the mitochondrial matrix. The reader is referred to other excellent reviews discussing other mitochondrial proteases^13,15,25,26^. We will discuss the molecular characteristics and biological roles of mitochondrial ClpXP and potential therapeutic strategies to target this protease for cancer therapy.

## Mitochondrial ClpP

**Fig. 2:**
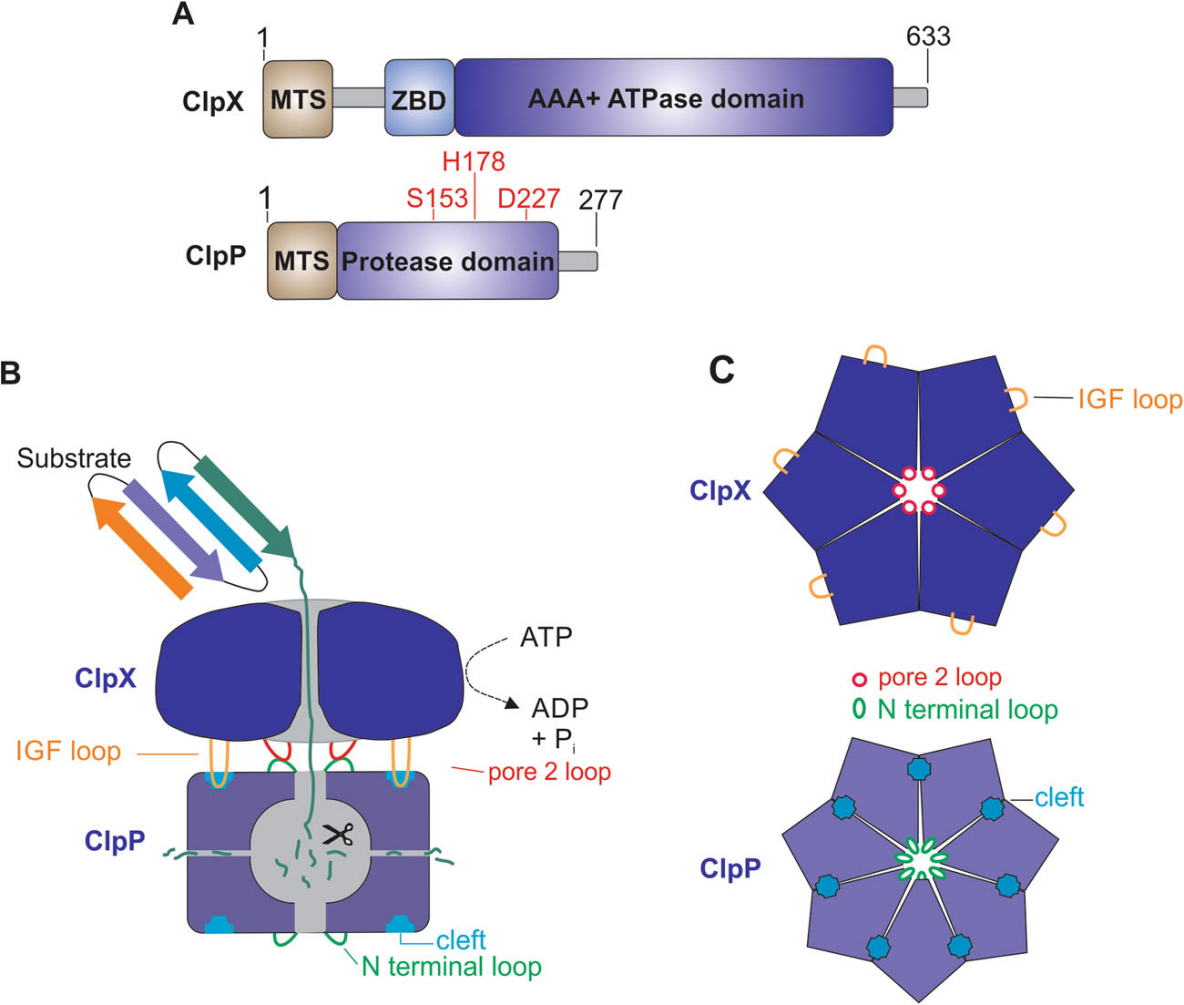
Structure and interaction of ClpP and ClpX. **a** Domain organization of ClpX (top) and ClpP (bottom) with catalytic residues of Ser153, His178, and Asp227. MTS mitochondrial targeting sequence, ZBD zinc-binding domain; AAA+, ATPases associated with diverse cellular activities. **b** Schematic representation of the ClpX and ClpP interaction and proteolytic cycle. **c** Top view of hexameric ClpX (top) and heptameric ClpP (bottom).

ClpP is located in the mitochondrial matrix of a diverse range of eukaryotes from C. elegans to human, although homologs are not found in yeast. In humans, ClpP is encoded on chromosome 19^27^. Once translated in the cytosol, it is directed to the mitochondrial matrix by a 56-residue N-terminal targeting sequence. This sequence is cleaved upon protein maturation in the mitochondrial matrix^1^. Mature human ClpP (hClpP) has 277 amino acids and shares high sequence similarity (71%) and identity (56%) with *E. coli* ClpP. However, mammalian ClpP, including the human homolog, has an extended 28 residues at its C-terminus (Fig. 2)^28,29^. This C-terminal extension forms an unstructured flexible loop which extends out of the surface of the oligomer. The role of this sequence is not well understood, but seems necessary for the stability of the protease, the assembly of the functional ClpP heptamer, and its affinity for its chaperone ClpX^28^.

Much of our understanding of the structure and function of human ClpP has been derived from studies of the bacterial homolog and the crystal structure of human mitochondrial at 2.1 Å (PDB: 1TG6)^28,30^. Similar to the bacterial enzyme, functional mitochondrial ClpP is a large cylindrical tetradecamer of two identical stable heptameric rings enclosing a large aqueous chamber.

Each ClpP monomer has a compact body, called the “head region”, and a unique expanded α/β unit called the “handle region”. Heads of seven monomers build up the heptameric rings through mostly hydrophobic interactions and the handles establish transient contacts to the adjacent heptameric ring via hydrogen bonds. The protease contains 14 internal catalytic cleavage sites and each subunit in the ClpP homotetradecamer has an active site with catalytic residues of Ser153, His178, and Asp227^2,28,30–32^.

Like bacterial ClpP, mitochondrial ClpP also has three different conformational states: extended, compacted, and compressed. Among them, only the extended form demonstrates catalytic activity required for substrate degradation, while the others are assumed to be part of a barrel-opening cycle^2,33–35^. Unlike bacterial ClpP that exists predominantly as a double-ring tetradecamer, human ClpP exists as an inactive but stable single heptamer ring under physiological conditions and in vitro^1,30,36^.

Mitochondrial ClpP lacks ATPase activity and each subunit contains only the domain for digestion of small peptides (six or fewer amino acids) without ATP requirement^37^. To have a processive proteolytic activity to degrade full length proteins, human ClpPs assemble into a tetradecamer in the presence of its ATPase, ClpX^1,10,30^.

## Mitochondrial ClpX complexes with ClpP to form an active protease

In mammalian cells, ClpP forms a heterodimer with its ClpX chaperone, forming a complex often termed ClpXP. ClpX is a member of the AAA+ protein superfamily (ATPases associated with various cellular activities). This nuclear-encoded protein is the only known ATPase component for mammalian ClpP^30^. However, in bacteria, ClpP can be complexed with ClpX, ClpC, ClpE, and others^1,38^. Like ClpP, human ClpX also contains an N-terminal 56-residue long mitochondrial targeting sequence (MTS) and shares 44% identity and 62% similarity with *E. coli* ClpX^1^. ClpX is a hexameric ring with 6-fold symmetry and is stabilized by binding ATP.

Assembly of the human ClpXP protease complex involves capping each end of the barrel-shaped ClpP tetradecamer with the ClpX hexamer^39,40^. How the ClpXP complex is formed in mitochondria is not fully understood, but based on studies on the bacterial ClpXP homologs it is likely that the interaction between ClpP and ClpX is stabilized by a tripeptide IGF loop on ClpX. This loop dynamically docks at a specific hydrophobic pocket on the apical surface of ClpP that is formed between every two ClpP subunits at the ClpX–ClpP interface^28,30,39^. Furthermore, the formation of the human ClpXP complex is mediated by interactions between the flexible N-terminal loop of ClpP and the pore-2 loop of ClpX (Fig. 2)^41,42^.

The substrate specificity of ClpXP is achieved by ClpX. Proteins destined for degradation by the ClpXP complex are recognized and unfolded by ClpX, which then fed into the lumen of ClpP’s proteolytic chamber and degraded into small peptides fragments and probably expelled through the transient side pores (Fig. 2). The unfolding and threading of proteins into ClpP by ClpX is an ATP dependent process, while the proteolysis of substrates by ClpP is independent of ATP hydrolysis^31,43^.

In bacteria, substrate recognition usually depends on specific linear sequence motifs located at either the N-terminus or C-terminus of the substrate named degrons^22,41,44^. These degrons can also be introduced by the specialized 11 residue SsrA peptide tagging system, which is used for rescuing stalled ribosomes^22,45^. Alternatively, substrate recruitment may be assisted by adapter proteins that tether selected substrate proteins to the Clp proteolytic complex, thus facilitating their degradation^44^. For instance, ClpXP can degrade substrates independently of adapters, but the adapter-like protein YjbH significantly enhances the proteolytic activity of the complex in *S. aureus*^46^. Likewise, the adapter MecA activates ClpC by targeting substrates and stimulating ClpC ATPase activity in *B. subtilis*^47^.

Structural differences between bacterial ClpX and mitochondrial ClpX mediate species-dependent differences in substrate specificities. Substrate recognition features of mitochondrial ClpXP and potential adapter proteins are unknown yet, and require further functional characterization.

## Cellular function of mitochondrial ClpXP

The main function of mitochondrial ClpXP is to maintain protein quality control by degrading denatured or misfolded proteins^48,49^. To date, several ClpXP substrates have been identified, including proteins involved in electron transport, metabolic processes, and the tricarboxylic acid cycle (TCA cycle)^29,49,50^. By degrading misfolded or damaged respiratory chain proteins, ClpXP maintains the integrity of the respiratory chain and sustains oxidative phosphorylation^49,50^.

ClpXP also regulates the mitochondrial unfolded protein response (UPRmt), a mitochondria-to-nucleus stress signalling pathway, which decreases mitochondrial translation, adjusts cellular metabolism, and supplies protection against pathogens^5,51^. Most of the information regarding ClpXP’s role in UPRmt is derived from studies in *C*. elegans, but a similar pathway is likely to present in higher organisms.

In *C. elegans*, ClpXP degrades unfolded or misfolded proteins in the mitochondrial matrix under protein folding stress or disruption of oxidative phosphorylation. Then, the produced small peptides are exported out of the mitochondria into the cytoplasm by the HAF-1 transporter^52^. Through a yet unknown mechanism, the efflux of these short peptides induces the expression of mitochondrial chaperones and proteases as part of a transcriptional response coordinated by activating transcription factor associated with stress 1 (ATFS-1). ATFS-1 contains both nuclear and mitochondrial target sequences. It is proposed that peptides generated by the enzymatic activity of ClpXP are exported from mitochondria and blunt ATFS-1 import into mitochondria. As a result, ATFS-1 is redirected to the nucleus where, along with ubiquitin-like 5 (UBL-5) and DVE-1 (Homeobox domain-containing protein), it induces the expression of various UPRmt target genes to maintain mitochondrial quality control and restore proteostasis^52,53^.

While better studied in *C. elegans*, less is known about the inducers of mammalian UPRmt. The mammalian homolog of HAF-1, has not yet been defined, but ATF5 is likely the ATFS-1 ortholog^52^. ClpXP is also likely an important mediator of mammalian UPRmt^49,54^.

In bacteria, the role of ClpX is confined to its function as a ClpP chaperone. However, in eukaryotes, including mammalian cells, mitochondrial ClpX has functions beyond its partner ClpP protease. For example, ClpX regulates heme biosynthesis in the mitochondria independent of ClpP. In a process conserved from yeast to mammalian vertebrates, ClpX, stimulates ALA (5-aminolevulinic acid) synthesis which is the first step of heme biosynthesis. ClpX (or the yeast homolog Mcx1) binds to ALA synthase (ALAS, Hem1 in yeast) and catalyzes the integration of the cofactor pyridoxal phosphate (PLP) into the ALA synthase apoenzyme, thereby generating an active form of ALA synthase and initiating heme biosynthesis^55,56^. In addition, by acting as a chaperone independent of ClpP, ClpX may stabilize the mitochondrial transcription factor A (TFAM) to regulate mtDNA nucleoid distribution^57^. Finally, a report by Verhagen et al., also discovered a role for ClpX in the cytoplasm, where it physically interacts with the XIAP (X-linked inhibitor of apoptosis) BIR2 domain to promote apoptosis^58^.

## Mitochondrial ClpXP and cancer

AML cells and stem cells, as well as subsets of other malignancies such as chronic myeloid leukemia (CML), pancreatic and breast cancer^49,59–64^ have unique mitochondrial characteristics with increased reliance on oxidative phosphorylation. The increased reliance on oxidative phosphorylation is due, at least in part, to increased flux of substrates into the TCA cycle^62,65^, decreased spare reserve capacity^61^, and an inability to upregulate other metabolic pathways upon inhibition of oxidative phosphorylation^59,65,66^. These data highlight a unique metabolic vulnerability and suggest that targeting oxidative phosphorylation could selectively kill these malignant cells. Targeting ClpXP is an emerging anticancer strategy that exploits the increased dependence of oxidative phosphorylation in these cancers.

To date, the majority of studies in cancer have focused on targeting ClpP and have not extensively investigated ClpX. For example, ClpP is overexpressed in subgroups of patients with multiple malignancies including acute myeloid leukemia^49^, breast, lung, liver, ovary, bladder, prostate, uterus, stomach, prostate, testis, thyroid, and non-small cell lung cancer (NSCLC)^50,67,68^. ClpP expression is positively correlated with UPRmt gene expression. However, the direct regulators of mammalian ClpP expression, such as transcription factors and epigenetic marks that lead to dysregulated expression in cancer have not yet been fully identified. In addition, ClpX expression in cancer has not been widely reported. Further studies are also required to determine how ClpXP contributes to the initial development of malignancy.

ClpP is necessary for the viability, growth, resistant, and metastasis of a subset of malignancies and inhibiting ClpP with genetic or chemical approaches kills malignant cells with high ClpP expression^49,50,68,69^. Consistent with its role in maintaining the integrity of respiratory chain complexes, loss of ClpP increases ROS production, decreases respiratory chain complex activity, impairs oxidative phosphorylation which appears functionally important for cell death after inhibiting ClpP^49,50^. While fewer studies have examined the importance of ClpX for the viability of malignant cells, it is believed that the results with ClpP are a surrogate for the activity of the ClpXP holoenzyme and inhibiting ClpX in cancer would produce similar results. However, this hypothesis needs to be tested experimentally.

In contrast to the cytotoxic effects of inhibiting ClpP in cancer, normal cells are relatively insensitive to loss or inhibition of ClpP. ClpP is predominantly expressed in tissues with high mitochondrial content such as skeletal muscle, liver, and heart. Despite its high expression in critical organs^27,70,71^, ClpP −/− mice are viable, but slightly smaller than their wild type counterparts. ClpP −/− are also infertile and acquire hearing loss^37^. In humans, rare individuals from consanguineous families have homozygous inactivating mutations in ClpP. These individuals are viable, but also have acquired hearing loss and infertility^72,73^. These studies support a therapeutic window for the development of ClpP inhibitors for the treatment of some malignancies. In addition, while other mitochondrial proteases are also potential anticancer targets, ClpP is unique in the relatively mild phenotype of the knockout mice and humans with ClpP mutations. In contrast, while inhibition of other proteases such as LONP1 kills cancer cells, homozygous deletion of *Lonp1* is embryonic lethal in mice^74^. The tolerability of ClpP loss in mice and humans also raises mechanistic questions as to why inhibiting ClpP is lethal to some cancers, but not normal cells with high ClpP expression.

## Small molecule ClpP inhibitors—chemical probes to understand ClpP biology and leads for anticancer agents

Small molecules that inhibit the proteolytic activity of ClpP have been developed as chemical probes to understand the biological functions of ClpP and further validate ClpP as a therapeutic target for malignancy. In 2008, activity-based protein profiling identified trans-ß-lactones as inhibitors of bacterial ClpP^75^. These ß-lactones attack the catalytic Ser of ClpP by its electrophilic core scaffold and covalently block the active site^76^. Crystal structure studies in *S. aureus* ClpP suggests the hydrophobic R1 chain of ß-lactones binds to a deep pocket adjacent to the ClpP active site^77^. This binding brings the ß-lactones core and catalytic Ser of ClpP into close proximity and promotes the nucleophilic attack^77^. Through their ability to inhibit ClpP, ß-lactones have antibacterial effects in vitro and in vivo.

In addition, one synthetic ß-lactone, A2-32-01, cross reacts with the mitochondrial ClpP enzyme^49^. A2-32-01 kills AML cell lines, and primary AML samples with high ClpP expression preferentially over normal hematopoietic cells and AML cells with low ClpP expression (Table 2 and Supplementary Fig. 1)^49^. A2-32-01 is also effective in mouse models of leukemia^49^. Although A2-32-01 is a useful chemical tool to study ClpP, its poor stability makes the compound unsuitable for clinical development as the cyclic ester of the ß-lactone is quickly hydrolyzed. In fact, more than 90% of A2-32-01 is hydrolyzed in cell culture media within 1 h^49^.

**Table 2:** Inhibitors and activators of mitochondrial ClpP.

Inhibitors					
Class	Name	Cell lines		Biological effect	Reference
ß-lactones	A2-32-01	TEX	Acute myeloid leukemia	Induced cell death	^49^
		OCI-AML2	Acute myeloid leukemia	Induced cell death; Reduced activity of respiratory chain complex II in SCID mice xenograft	^49^
		K562	Chronic myeloid leukemia	Induced cell death	^49^
		HL60	Promyelocytic leukemia	No effect on cell viability	^49^
		143B	Osteosarcoma	Induced cell death	^49^
		143B Rho (0)	Mitochondria depleted osteosarcoma	No effect on cell viability	^49^
Phenyl esters	AV167	N/A	N/A	N/A	^78^
	TG42	Huh7	Hepatocyte-derived carcinoma	Induced cell apoptosis Decreased cell migration	^79^
		Jurkat	Human T lymphocyte	Target a range of human proteases including ClpP	^79^
	TG53	Huh7	Hepatocyte-derived carcinoma	Induced cell apoptosis Decreased cell migration	^79^
α-aminoboronic acid	8a	N/A	N/A	N/A	^81^
	8b	N/A	N/A	N/A	^81^
	8c	N/A	N/A	N/A	^81^

Activators					
Class	Name	Cell lines		Biological effect	Reference
ADEP	ADEP-41	HEK293 T-REx	Embryonic kidney cells	Induced mitochondrial fragmentation; abolished OXPHOS function and induced apoptosis	^89^
		HEK293 T-REx ClpP−/−	Embryonic kidney cells with ClpP knock out	No effect on cell viability; no change in mitochondrial morphology	^89^
		HeLa	Servical carcinoma	Induced cell death	^89^
		HeLa T-Rex	Cervical carcinoma	Induced cell death	^89^
		U2OS	Osteosarcome	Induced cell death	^89^
		SH-SY5Y	Neuroblastoma	Induced cell death	^89^
Imipridones	ONC201	TEX	Acute myeloid leukemia	Reduced growth and viability	^82^
		OCI-AML2	Acute myeloid leukemia	Impaired respiratory chain complexes I, II, and IV; reduced growth and viability; reduced the leukemic burden in mice	^82^
		OCI-AML3	Acute myeloid leukemia	Decreased respiratory chain complex protein levels; damaged mitochondrial matrix and cristae structures; reduced growth and viability	^82^
		Z138	Mantle cell lymphoma	Decreased respiratory chain complex protein levels and oxygen consumption rate; increased ROS prodcution; reduced growth and viability	^82^
		HEK293 T-REx	Embryonic kidney cells	Reduced cell viability	^82^
		HEK293 T-REx ClpP−/−	Embryonic kidney cells with ClpP knock out	No effect on cell viability	^82^
		HCT-116	Colorectal carcinoma	Reduced growth and viability	^82^
		HeLa	Cervical carcinoma	Reduced growth and viability	^82^
		OC316	Ovarian serous adenocarcinoma	Reduced growth and viability	^82^
		SUM159	Pleomorphic breast carcinoma	Reduced growth and viability	^93^
		MDA-MB-231	Breast adenocarcinoma	Reduced growth and viability	^93^
	ONC212	TEX	Acute myeloid leukemia	Reduced growth and viability	^82^
		OCI-AML2	Acute myeloid leukemia	Reduced growth and viability	^82^
		OCI-AML3	Acute myeloid leukemia	Decreased respiratory chain complex protein levels; reduced growth and viability	^82^
		Z138	Mantle cell lymphoma	Reduced growth and viability; mice xenograft had decreased tumor burden and prolonged lifespan	^82^
		HCT-116	Colorectal carcinoma	Decreased respiratory chain complex protein levels; reduced growth and viability	^82^
		HeLa	Cervical carcinoma	Decreased respiratory chain complex protein levels; reduced growth and viability	^82^
		OC316	Ovarian serous adenocarcinoma	Decreased respiratory chain complex protein levels; reduced growth and viability	^82^
		SUM159	Pleomorphic breast carcinoma	Decreased respiratory chain complex protein levels; reduced growth and viability	^82^
	TR57	SUM159	Pleomorphic breast carcinoma	Reduced growth and viability; induced ATF4 and activated integrated stress response	^93^
		MDA-MB-231	Breast adenocarcinoma	Reduced growth and viability; induced ATF4 and activated integrated stress response	^93^

A screen of over 137,000 compounds identified phenyl esters as inhibitors of bacterial ClpP peptidase activity. Like ß-lactones, phenyl esters inhibit ClpP through a nucleophilic attack on the catalytic Ser residue^78^. The ester is cleaved, thus trapping ClpP in the acyl-enzyme intermediate state and consequentially causing the deoligomerization of ClpP^78^. Five phenyl ester compounds AV126, AV168, AV127, AV167, and AV170 were identified that have improved potency, kinetics, and stability against bacterial ClpP compared with ß-lactones^78^. Interestingly, despite the significant homology between bacterial and human ClpP only AV167 cross reacts with human ClpP (Table 2 and Supplementary Fig. 1)^78^, suggesting important differences in the active sites between human and bacterial ClpP. Through substitutions of the naphtofuran moiety at position-2 of AV167, more potent and selective inhibitors of human mitochondrial ClpP were developed^79^. The modified analogs, termed TG42, TG43, and TG53, preferentially inhibit human ClpP’s peptidolytic and proteolytic activities while having a minor effect on *S. aureus* ClpP (SaClpP)^79^. TG42 and TG53 induce apoptosis and decrease cell migration of Huh7 liver cancer cells (Table 2 and Supplementary Fig. 1)^79^. However, further studies are necessary to determine whether these anticancer effects are due to ClpP inhibition or off-target effects as these compounds cross react with multiple human proteases.

Peptide boronates were also identified as ClpP inhibitors^80^. From this class, α-aminoboronic acids compounds **8a**–**c** were identified as human ClpP inhibitors with comparable potency with AV167^81^. Virtual modeling of α-aminoboronic acid with human ClpP suggests that the compound interacts with Ser97 and H122 of human ClpP^81^, but physical structures would be necessary to confirm the mechanism of inhibition. In addition, the ability of the compounds to bind and inhibit ClpP in the intact cell needs to be assessed.

To date, efforts to target ClpXP have focused on inhibiting the active site of ClpP. However, compounds that disrupt the interaction between ClpP and ClpX could be novel inhibitors and might have improved selectivity for the target. For instance, the highly conserved IGF motifs and the pore-2 loops of ClpX, which represents two sets of well-characterized interaction points between ClpX and ClpP could potentially be targeted to interrupt the interaction and thereby inhibit ClpXP. In addition, molecules that block the ATPase function of ClpX could also be novel anticancer agents.

## ClpP hyperactivation—biology and anticancer effects

ClpP is a unique cancer target as both inhibition and hyperactivation kill malignant cells, although through different mechanisms. Inhibiting ClpP leads to the accumulation of damaged and misfolded respiratory chain proteins that impairs oxidative phosphorylation and causes cell death. In contrast, hyperactivating ClpP leads to uncontrolled, but selective, degradation of ClpP substrates including respiratory chain proteins. As a result, hyperactivation of ClpP leads to decreases in levels of respiratory chain proteins that also impairs oxidative phosphorylation and causes cell death^82^.

Small molecules that hyperactive ClpP have been identified. In contrast to ClpP inhibitors that mainly target the catalytic triad of the serine protease, ClpP activators displace ClpX, open the pore of the ClpP protease, and thereby increase its protease activity. Similar to inhibitors of mitochondrial ClpP, the development of mitochondrial ClpP activators also started from studies with the bacterial homolog. Acyldepsipeptides (ADEPs) are a class of antibiotics with an unknown mechanism that were initially isolated from the fermentation broth of *Streptomyces hawaiiensis*^83^. Later, bacterial ClpP was determined as the molecular target of ADEP through a genomic analysis of ADEP-resistant *E. coli*.^84^. ADEPs bind to bacterial ClpP at hydrophobic pockets (H pockets) and destabilize the N-terminal of ClpP, thereby displacing the regulatory subunits such as ClpX and opening the entry pore of ClpP^85^. As a result, ADEP–ClpP complex has increased proteolysis of cell division protein FtsZ, nascent polypeptide chains, transcriptional factors MecA, and other key regulators, resulting in bacterial cell death^84,86–88^.

Given the cytotoxicity of ADEPs for bacterial ClpP, their effects on mitochondrial ClpP were explored. ADEP and ADEP analogs also bind human ClpP at H pockets and cause displacement of ClpX and activation of the protease^89^. In malignant cells, an ADEP analog, ADEP-41, disrupted mitochondrial function and caused cell death (Table 2 and Supplementary Fig. 1)^89^.

The imipridone family is another family of anticancer compounds recently identified as ClpP activators^82^. ONC201 is the first-in-class imipridones that is in clinical trials for multiple advanced cancer. Although initially was thought to antagonize dopamine D2 receptors and activate the integrated stress response, these actions cannot fully explain the mechanism of action of these drugs^90–92^. More recently, the imipridones, including ONC201, were shown to bind and activate human ClpP^82,93^. Imipridones activate ClpP through the same mechanisms as ADEP but with a higher potency. The co-crystal structure shows seven ONC201 molecules occupy the hydrophobic pockets of ClpP leading to compaction of the protease and opening of the axial pore^82^. ONC201 and ONC212 kill malignant cells including primary samples from AML patients in vitro and in vivo (Table 2 and Supplementary Fig. 1). In addition, malignant cells with the highest levels of ClpP are most sensitive to these compounds.

Mechanistically, these compounds decrease respiratory chain complex proteins, impair respiratory chain complex activity, and increase ROS production. Increased expression of UPRmt proteins was observed, but further studies are necessary to determine how activation of UPRmt contributes to cell death after imipridones treatment.

Activation of ClpP by imipridones is functionally important for their cytotoxicity as the compounds are inactive in cells with mutated or depleted ClpP.

Imipridones are also involved in other cellular activities including activation of the intergraded stress response, inhibition of mTORC1 pathway, and Akt/ERK inactivation^90,92^. Since imipridones are also reported to antagonize dopamine D2 receptors (DRD2) and activate orphan G protein-coupled receptor GPR132, future studies may elucidate which of these effects are mediated by ClpP and what are due to targets beyond ClpP, such as DRD2 and GPR132 receptors^91,94^.

Whether ClpP inhibition or ClpP hyperactivation is a more efficient strategy to target cancer cells is a critical question which needs to be answered in future studies.

## Concluding remarks and future directions

Emerging evidence indicates that mitochondrial ClpXP is necessary for a subset of hematologic malignancies and solid tumors. These studies in cancer cells have highlighted ClpXP as a novel therapeutic target, but also provide important insight into the normal function of this mitochondrial protease and mitochondrial metabolism. Unique to this protease, both inhibition and hyperactivation of ClpP impair oxidative phosphorylation and have anticancer effects.

Recent studies have identified several classes of molecules that target and modulate ClpP proteolytic activity with different degrees of selectivity and specificity. While these compounds may represent promising new approaches to selectively target cancer, more research is required to optimize their potency, stability, and selectivity. Moreover, additional studies are required to better characterize their in vivo efficacy and toxicity. Fortunately, virtual and physical structures of ClpP are available to guide these studies.

In the context of developing clinical grade molecules that target ClpP, biomarkers to identify populations of patients most and least likely to respond should also be developed. In some malignancies, such as AML, levels of ClpP correlate with response to ClpP targeted therapies in vitro. As such, ClpP protein expression could be developed as a future biomarker to predict response and select patients for therapy. However, the impact of chemotherapy on ClpXP expression and if expression of ClpXP changes at relapse need to be addressed.

In addition, it will be important to understand the mechanism of resistance to ClpP inhibitors and activators and identify the strategies to overcome them. Finally, further understanding the mechanism of action of ClpXP and related proteases remains critical, both for our ability to translate new therapies to the clinic as well as to understand mitochondrial biology.

## Supplementary information

Supplementary information

Supplementary Figure 1
