# Supplementary material for: Mitochondrial ClpP serine protease-biological function and emerging target for cancer therapy
Source: Cell Death Dis. 2020 Oct 9;11(10):841. doi: 10.1038/s41419-020-03062-z (PMC7547079; doi:10.1038/s41419-020-03062-z)

**Mitochondrial ClpP Serine Protease -Biological Function and Emerging Target for Cancer Therapy**

Kazem Nouri,^1,3^ Yue Feng,^1,2,3^ Aaron D. Schimmer^1,*^

***Supplementary Figure 1. Chemical modulators of mitochondrial ClpP.***

(A) Inhibitors of ClpP. (B) Activators of ClpP.


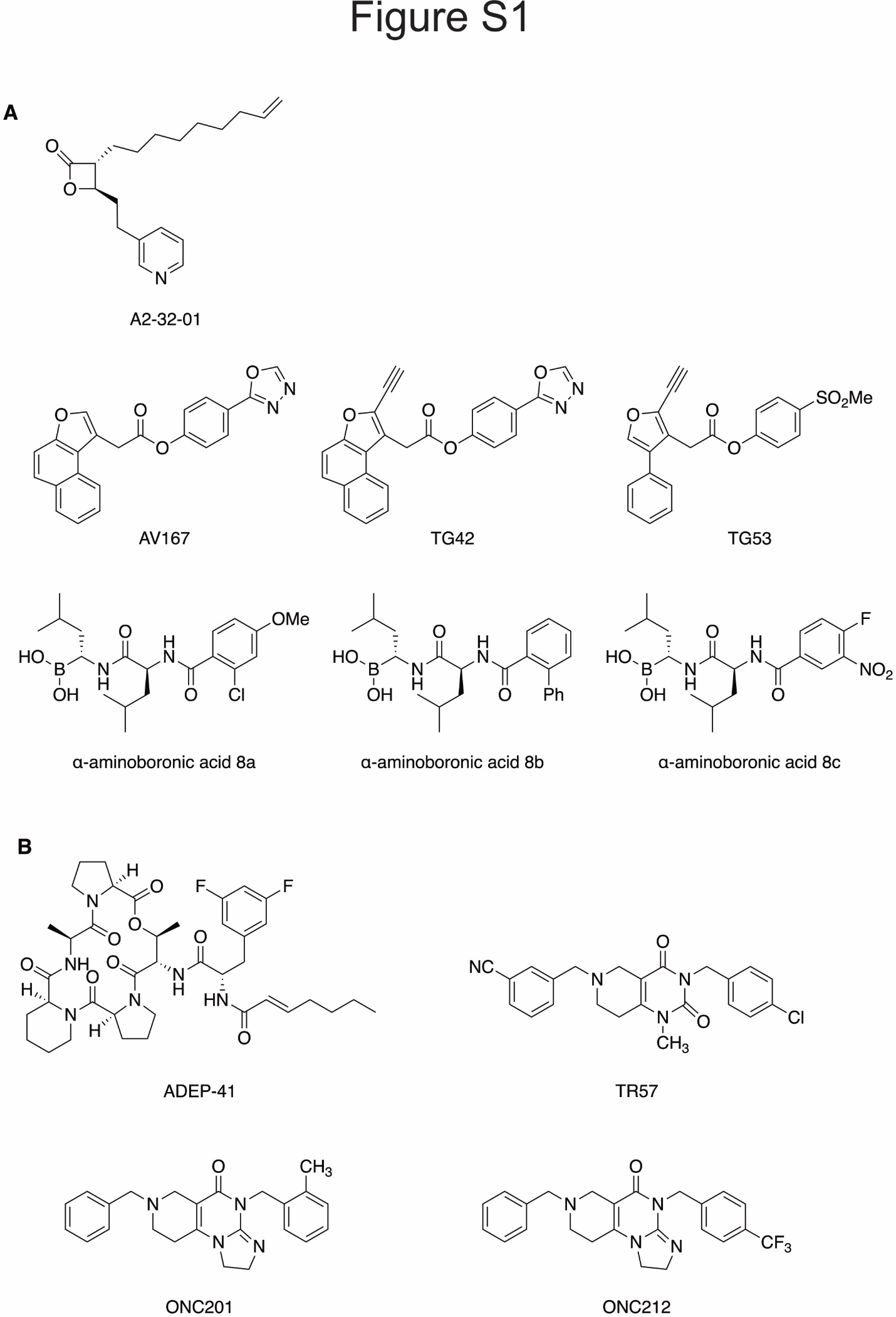

Supplement: Supplementary file 1 — Supplementary information [file 41419_2020_3062_MOESM1_ESM.docx]
