# Supplementary figures and images for: Mitochondrial ClpP serine protease-biological function and emerging target for cancer therapy
Source: Cell Death Dis. 2020 Oct 9;11(10):841. doi: 10.1038/s41419-020-03062-z (PMC7547079; doi:10.1038/s41419-020-03062-z)

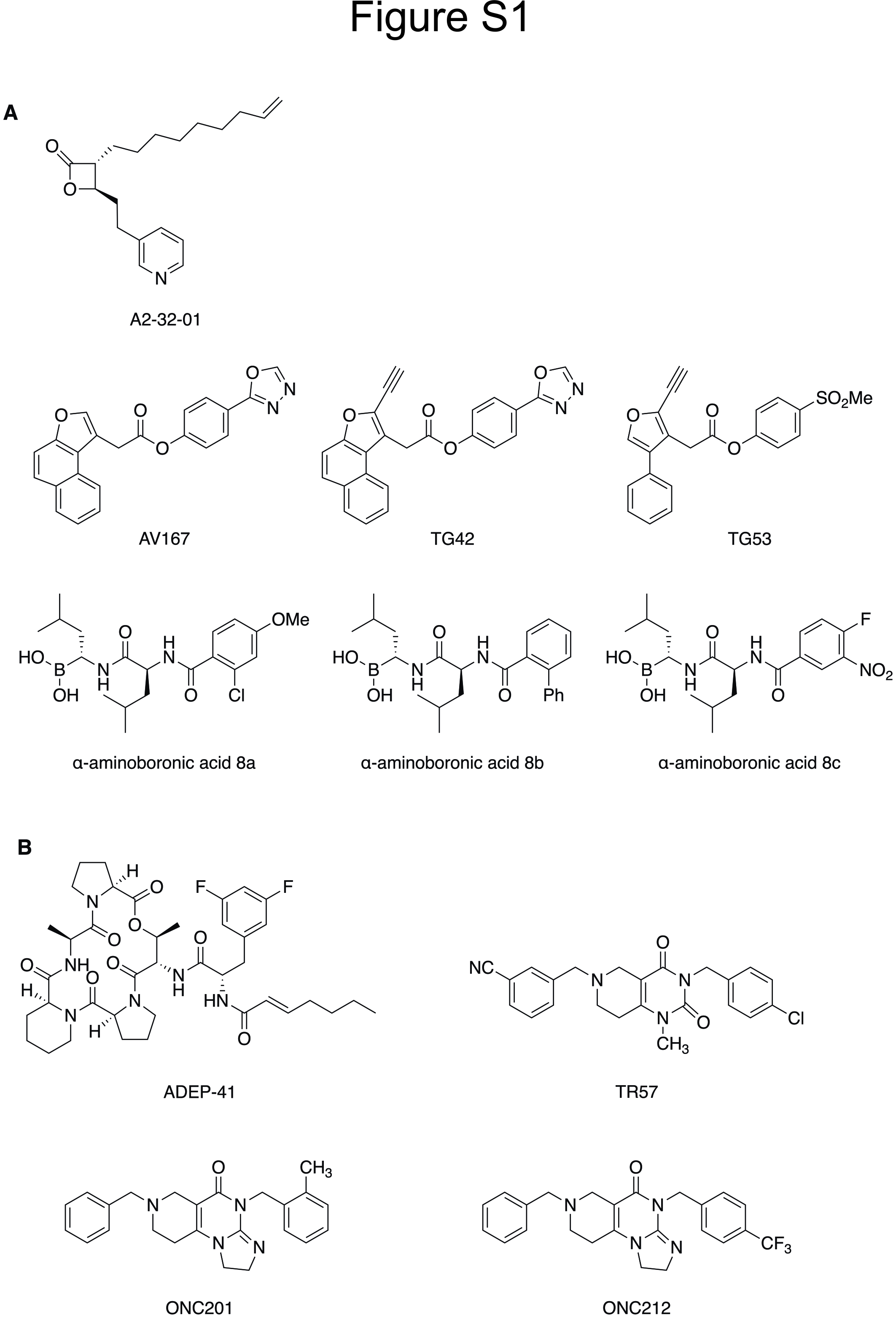

Supplement: Supplementary file 2 — Supplementary Figure 1 [file 41419_2020_3062_MOESM2_ESM.jpg]
